# Recent advances in targeting LRRK2 for Parkinson’s disease treatment

**DOI:** 10.1186/s12967-025-06354-0

**Published:** 2025-07-08

**Authors:** Mahsa Karami, Pantea Majma Sanaye, Atousa Ghorbani, Roshanak Amirian, Pouya Goleij, Mehregan Babamohamadi, Zhila Izadi

**Affiliations:** 1 Student Research Committee, Kermanshah University of Medical Sciences, Kermanshah, Iran; 2 Pharmaceutical Sciences Research Center, Health Institute, Kermanshah University of Medical Sciences, Kermanshah, Iran; 3 USERN Office, Kermanshah University of Medical Sciences, Kermanshah, Iran; 4 School of Pharmacy, Zanjan University of Medical Sciences, Zanjan, Iran; 5 Department of Biology, North Tehran Branch, Islamic Azad University, Tehran, Iran; 6 Department of Genetics, Sana Institute of Higher Education, Sari, Iran; 7 Department of Biology, School of Natural Sciences, University of Tabriz, Tabriz, Iran

**Keywords:** Parkinson’s disease, LRRK2, Neurodegenerative diseases, Autophagy, PROTAC, Mitochondrial dysfunction, Neuro-inflammation

## Abstract

Parkinson’s disease (PD) is a neurodegenerative disease with severe movement problems. Current treatments mainly focus on symptom management by reducing dopaminergic pathways in the brain. Despite these therapies, ongoing disease progression undermines the effectiveness of prevalent approaches, necessitating exploring alternative methods anchored on genetic factors, notably the leucine-rich repeat kinase 2 (LRRK2) gene. Exploring LRRK2 gene pathogenesis has highlighted various mechanisms that may contribute to treating PD, including protein accumulation, altered cytoskeletal dynamics, neuro-inflammation, autophagy, and mitochondrial dysfunction. Based on the findings, there is an actual correlation between elevated levels of LRRK2 and the biomarkers and assays of PD. Furthermore, research results have suggested inhibiting LRRK2 as a therapeutic intervention targeting pathogenic mechanisms with varying degrees of efficacy. Our review wants to understand how LRRK2 works in the body and its relationship with the occurrence of PD by providing biochemical evidence, LRRK2 gene mutations and pathology, and the role of this gene in the immune system. We also discuss targeted therapies such as kinase inhibitors and Proteolysis targeting chimera and the application of using the LRRK2 protein to diagnose PD and develop bioassay designs. Finally, we mention the clinical trials conducted and the challenges and safety required.

## Background

Recent research has turned its attention to genetic factors implicated in PD, with the leucine-rich repeat kinase 2 (LRRK2) gene emerging as a significant player. Mutations in the LRRK2 gene are among the most common genetic causes of PD, and understanding the pathogenesis associated with these mutations is crucial for developing new therapeutic strategies. Studies have identified several mechanisms through which LRRK2 may contribute to PD, including protein aggregation, disrupted cytoskeletal dynamics, neuroinflammation, impaired autophagy, and mitochondrial dysfunction. This review aims to elucidate the role of LRRK2 in PD by examining biochemical evidence, gene mutations, and the gene’s involvement in the immune system. Additionally, it explores targeted therapies, including kinase inhibitors and Proteolysis Targeting Chimeras (PROTACs), and discusses the potential of using LRRK2 as a biomarker for PD diagnosis and bioassay development. The review also addresses the current state of clinical trials and the challenges and safety considerations associated with LRRK2-targeted therapies.

## Introduction

Parkinson’s disease (PD), the second common rationale for dementia, is a neurodegenerative disease affecting over 10 million people worldwide, targeting their motor abilities and other nervous functions [1]. No apparent racial difference is seen in PD’s occurrence, but it is more common in men than women [2]. In 2020 investigations, PD was responsible for a staggering healthcare burden of approximately $24,000 per individual [3]. PD’s yet unclear underlying pathogenesis and non-efficient treatments, besides its chronic progressive nature, demand us to be prepared for more to pay. With dominant motor symptoms, spotlights have long been brought on voluntary control areas in the brain, basal ganglia, and insufficient dopaminergic pathway [1]. However, prodromal symptoms and lack of reliable and sensitive biomarker progression have made us look elsewhere for therapeutic alternatives [4]. One of the primary risk factors in familial and sporadic PD, which leads to many pathological dysfunctions, is the mutation in the gene encoding leucine-rich repeat kinase 2 (LRRK2). The studies authenticated that the mutation rate is about 5–6% in familial and 1–2% in sporadic PD patients [5]. LRRK2 is a complex multifunctional protein with a few roles defining genetic areas like a Ras-of-complex (Roc) domain plus a C-terminal (COR) domain (ROC-COR GTPase), a mitogen-activated protein kinase (MAPK), and WD40 domains (WDR), which gives researchers a vast field of novel approaches to tackle this considerable healthcare burden [6, 7]. In the present study, we try to weigh up the probable horizon of LRRK2 in understanding PD’s pathology and drug discoveries.

## Parkinson’s disease

PD, the second neurodegenerative disease after Alzheimer’s, causes movement disorder and is likely for a neurologist’s daily encounters with patients. PD ensues because of selective neuron degeneration and loss of dopamine (DA) neurons in the substantia nigra pars compacta (SNPc). The main pathological hallmark of PD is α-synuclein (aSyn) inclusions, named Lewy bodies [8, 9]. Better control of the symptoms would be possible with an earlier diagnosis [10, 11], some imaging techniques could be helpful for the diagnosis of PD, but it is not complicated to clinically diagnose PD with its primary motor symptoms such as tremors, rigidity, bradykinesia/akinesia, postural instability, also non-motor symptoms including rapid eye movement, sleep disorder, hyposmia, constipation, cognitive problems (depression, anxiety) [8, 9]. The current approach for treating PD leans on the dopaminergic pathway, decreasing symptoms and decreasing the progression, but it is not curative [11, 12]. Today’s treatments are moving toward neurotrophic agents, electrical stimulation, cell therapy, and other novel options on track, alongside current strategies of medications, rehabilitation, and surgeries [13].

## LRRK2 and Parkinson’s disease

**Fig. 1:**
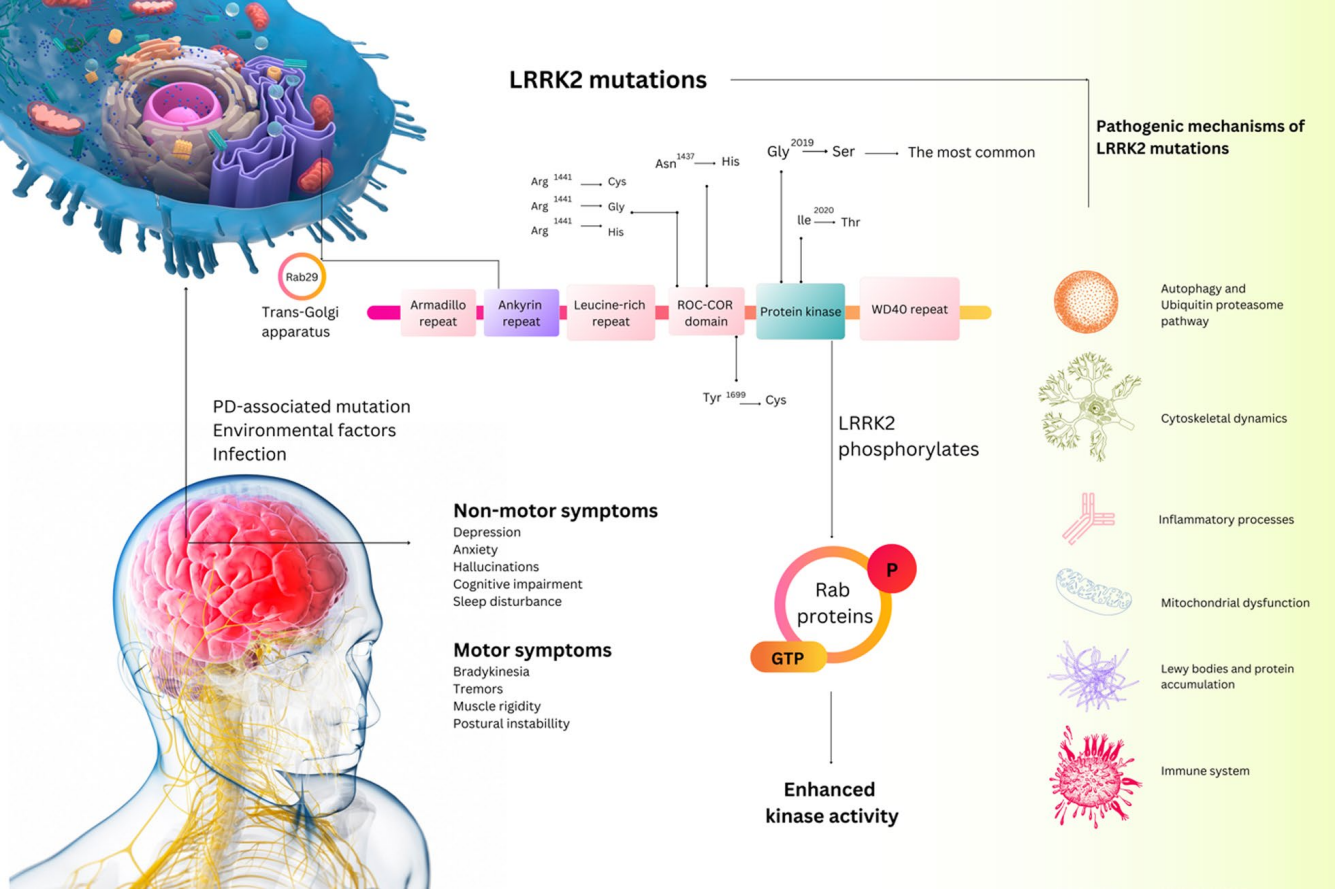
Illustration of human LRRK2 protein with presently recognized pathogenic mutations linked to PD leads to increased kinase activity. LRRK2 pathologies on Autophagy and Ubiquitin proteasome, pathway, cytoskeletal dynamics, mitochondrial dysfunction, Lewis bodies, and the immune system

Genetic risk factors and recessive and dominant genetic mutations have been specified in families with a high PD (fPD) prevalence, accounting for approximately 10–15% of all PD cases [14, 15]. No cure for PD would halt its progression or reverse it [10, 16]. Some markers could indicate PD, but there are currently no precise tests or biomarkers in clinical use that would enable the disease to be detected earlier before symptoms appear [10]. The most frequently suggested method for diagnosing the disease is molecular testing in all instances [17]. The various forms of PD caused by several underlying causes further complicate diagnosis and treatment, rendering some treatments ineffective [16]. Numerous monogenic forms of PD and several genetic risk factors that increase the likelihood of developing neuron degeneration have been identified through PD genetic research [18]. Studies have exhibited that there is a potent genetic association, with the mutation in 6 genes as the leading cause: pink1 (PTEN-induced kinase 1), dj1 (DJ-1), vps35 (Vacuolar protein sorting ortholog 35), snca (aSyn), and LRRK2 (Fig. 1) [19, 20]. Autosomal dominant missense mutations in the LRRK2, uncovered in the locus of chromosome 12q12 termed PARK8, are a primary cause of inherited PD, and therapeutic efficacy related to the LRRK2 inhibitors in clinical trials is being tested [21, 22]. Patients with PD-associated LRRK2 mutations exhibit varying symptoms across families and even within families with the same mutation [23]. LRRK2 mutations, especially G2019S, which increase kinase activity and are in the kinase domain, are also exciting because of their high incidence in PD patients [22]. Also, G2019S-LRRK2 mutation not only reported contributing to approximately 1–5% of sporadic PD cases but also reported in familial PD [24–27]. The product of this gene is a large protein of approximately 286 kDa named Dardarin [28, 29]. Mutations strongly influence the activity and conformation of Dardarin proteins in the LRRK2 gene. Several studies illustrated that Dardarin mutants could trigger programmed cell death, and interaction with parkin increases the level of cytoplasmic proteins [30]. As a result, LRRK2 has emerged as a crucial player in PD’s sporadic and common pathogenesis. Understanding the exact function of LRRK2 may assist in comprehending PD’s progression. Targeting the LRRK2 is essential for PD associated with LRRK2 dysfunction and is also expected to help treat PD that other gene mutations or agents cause.

## Biochemical evidence of LRRK2

Most LRRK2 studies have focused on missense mutations and mainly if/how these pathogenic mutations affect on enzymatic functions of proteins; the G2019S mutant of LRRK2 is located in the kinase domain activation loop and has a significant effect on the kinase activity of LRRK2, leading to a twofold increase [31–33]. Investigation in recent years has clarified several characteristics of LRRK2 function, like acting as a kinase that autophosphorylates itself at multiple serines and threonines in or near its ROC domain, including at Ser1292. The Ser1292 phosphorylation is evaluated as identification for LRRK2 autophosphorylation in vitro and in vivo. Studies explained that all comprehended pathogenic LRRK2 mutations enhance the enzyme’s kinase activity [34–37]. The Rab proteins family are GTPases that act as pilot regulators of vesicle formation, fusion, and trafficking. As a pivotal factor, Rab GTPase participates in protein transport and autophagy and plays a critical role in membrane vesicular trafficking in eukaryotes [37–42]. The pathogenic mutations in the ROC-COR domain (Y1699C, R1441H, R1441G, and R1441C) have a considerable effect on kinase activity than G2019S, which is reported to influence fourfold plenty in in-vivo investigations, which may be concluded that the mutation in the Roc and COR domains makes LRRK2 more effectively conscripted to the membrane-bound compartments Rab proteins, which exert proper allosteric regulation on LRRK2 functioning [36, 37, 40, 43–46]. However, conflict was observed in different studies. For instance, deficiency of the direct correlation between in-vivo and in-vitro kinase activity of LRRK2 readouts is demonstrated via the G2385R risk factor, which has declined in LRRK2 kinase activity when evaluated in-vitro, not in-vivo situation operating Rab10 protein as a substrate [46–48]. Subsequently, despite the mechanical complexities, it is precise that pathogenic mutations naturally occur and elevate LRRK2 kinase activity which potently affects kinase activity as a primary effector of the LRRK2-mediated pathway in different pathobiology.

## LRRK2 biology, structure, and mechanism

**Fig. 2:**
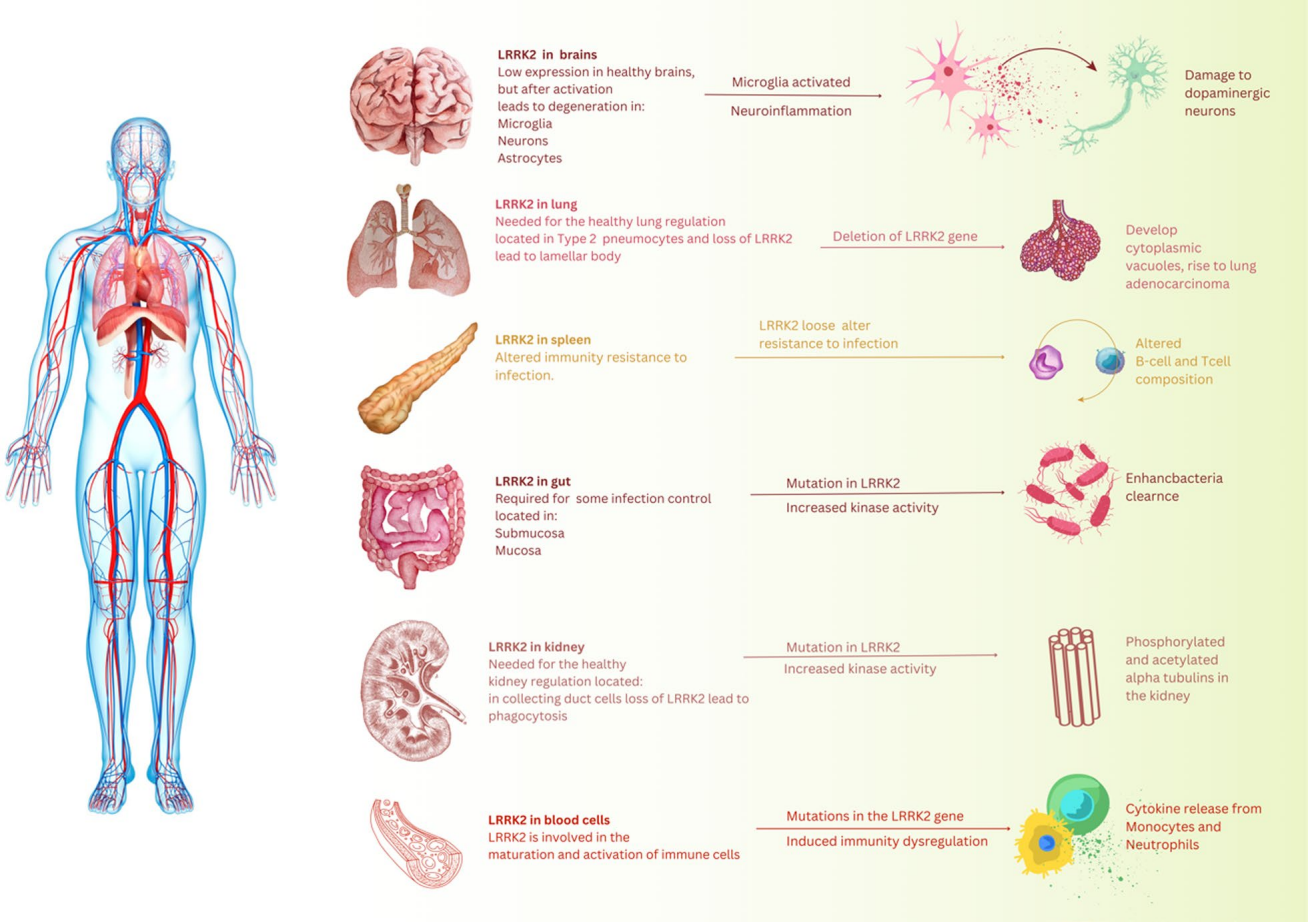
Illustration of LRRk2 expression in different tissue and cell types. In the healthy brain, LRRK2 has expressed at low levels in microglia. In contrast, PD brains observed increased expression levels in LRRK2 and activated microglia, leading to neuroinflammation and dopaminergic neuron death. Despite being associated with gut inflammation, LRRK2 is required for spleen, lung, and kidney health. LRRK2 expression is increased in peripheral immune cells in PD patients leading to increases in cytokine release from monocytes and neutrophils

Studies have verified that besides the brain, including the dorsal striatum (caudate and putamen), which receives a dense dopaminergic innervate, LRRK2 is expressed in several human organs, including the kidneys, liver, heart, spleen, and lungs (Fig. 2) [49–54]. Correspondingly to the brain, the study showed that the LRRK2 expression level is exceptionally high in the CNS of mice, especially in the striatum’s putamen, but after birth, expression levels of LRRK2 deluge [55–57].

Since LRRK2’s first discovery, considerable investigation has been accomplished into understanding its structure and function in different tissues. Attending the LRRK2 role is defined in PD and recreates a crucial standing in an appealing therapeutic target. LRRK2 is an enormous, complex protein (∼286 kDa, 2527 amino acids) with two middle enzymatic domains: a kinase domain and an ROC (Ras of complex proteins) domain that attaches GTP. Between the ROC and kinase domains is the COR (C-terminus of ROC) domain, which assembles LRRK2 dimers and regulates the GTP binding [29, 58]. LRRK2 is a serine-threonine kinase that can phosphorylate several downstream substrates [37, 44, 59–61]. The Rabs (Rab3D/C/B/A, Rab8B/A, Rab43, Rab12, Rab35, Rab29, and Rab10), recognized as physiological substrates of LRRK2 and repeatedly employed in both in-vivo and in-vitro as verified readout characteristics of LRRK2 kinase activity [60, 62–64].

The much conserved RocCOR tandem domain controls LRRK2’s GTPase activity or GTP hydrolysis. RocCOR domain data from prokaryotes suggest LRRK2 accomplishment as a GAD enzyme (G proteins activated through guanine nucleotide-dependent dimerization) which the COR domain acts as the mediating factor through the LRRK2 structure that promotes homo-dimerization, the LRRK2 switches between dimeric and monomeric (inactive form and primarily located in the cytosol with reduced kinase activity) in a GTP/GDP pendant mode, but the precise mechanism of LRRK2 GTPase activity is even up for controversy [65–71]. Prior studies have indicated biochemical and genetic interactions between Rab29 and LRRK2. Rab29 is the exclusive one connected to PD because of encoding and the PARK16 locus. In addition, the activation of LRRK2 kinase is controlled via Rab29 as one of the kinase substrates [45, 72, 73]. Studies showed that members of the regulatory 14-3-3 proteins family also bind to LRRK2 by protein kinase A (PKA), resulting in negatively regulating the kinase activity of LRRK2 [74, 75].

Furthermore, various LRRK2 mutations induce the 14-3-3 proteins’ binding missing, including I2020T, Y1699C, and R1441G, correlated with aberrant hyperactivity in the kinase role of LRRK2. As a result, LRRK2’s dimerization, GTPase domain activity, and interactions with other regulatory proteins influence its activation. However, additional research is required to provide a complete picture because it still needs to be determined whether other protein–protein interaction domains of LRRK2 are present in the activation [76].

## LRRK2 gene mutation

To date, 19 PARK loci have been designated for different genetic forms of PD, and the underlying gene mutation has been approved in 11 of them, with some uncertainty about the accuracy of the assignment of several genes in four loci [77–84]. Subsequent meta-analyses have confirmed these associations and the most recent GWAS [85–87]. Likewise, seven of the approximately 100 mutations found in this gene so far have been conclusively identified as causing disease: I2020T, G2019S, Y1699C, N1437H, and G2019S [88]. The two most missense common mutations, R1441C and G2019S, account for over 30% of inherited PD cases in some communities and 10 and 2.5% of sporadic PD patients [89, 90]. Sporadic mutations are probably due to the introgression of age-related mutations [91, 92]. Moreover, R1441C showed maintaining less penetrance, signifying that even these monogenic mutations considerably raise disease chance, but it should be mentioned that necessarily, in every case, leads to disease [93]. Further, the mutation in MAP kinase (MAPK) domain G2019S is mainly known due to late-onset LRRK2-associated PD and raises Vmax for kinase activity, whereas the Roc domain-specific Y1699C and R1441C/G/H mutations lower GTPase activity [94]. Because the GTPase activities and kinase encoded on the same protein may control one another, it is hypothesized that these two events are connected. Measures of Rab substrates in vitro support this idea that all mutations favor increased phosphorylation [14, 45, 61, 81, 95]. Numerous studies confirmed the twofold risk increase, establishing G2385R as a risk factor in Asian populations [96, 97]. This mutation, which occurs in the C-terminal WD40 domain, is likely responsible for altering the protein’s structure and influencing LRRK2’s attached to vesicular trafficking interactors [98]. Recent data suggest that the G2385R variant expresses lower steady-state intracellular protein levels than wild-type LRRK2. This is because, mechanistically, G2385R has a greater affinity than the wild-type protein for two proteasomal degradation proteins, the CHIP and Hsc70 [99, 100]. As a result, this mutation compromises the LRRK2 dimerization [101]. Description of how it affects kinase activity, clarified protein can reverse the G2019S mutation’s hyperactivation effect by reducing autophorylation in in-vitro kinase assays [46, 99, 101]. The G2385R LRRK2 variant is sequestered to the TGN, similar to the WT protein. The G2385R risk variant exhibits increased kinase activity toward Rab10 compared to WT protein in cell co-expression investigations, just like the further genetic variant [100, 102]. These findings suggest that familial mutations may activate some molecular pathways that moderate G2385R disease risk; regardless, the consequences of this variant initiate G2385R as a risk factor instead of a penetrant mutation. [103]. Intriguingly, the R1398H variant has been identified by decreasing the activity of the protein in several cohorts as protective against PD [104, 105]. Six studies from Greece [106], China [107], Norway [108], Zambia [109], Kazakhstan [110], and Sweden [111] sought to identify the N1437H variant PD in 3,368 PD cases [24]. These results demonstrate that non-coding and coding variants at the LRRK2 locus influence penetrance, onset age, and prevention of PD expansion. Furthermore, the cases are called "sporadic" PD, indicating that LRRK2 is more involved in the predominant disorder. Another way to consider the role that LRRK2 plays in the pathobiology of PD is that because various alleles have different penetrations, some will appear in families in a recognizable progression. In contrast, others will only appear in isolated cases with no family history.

### LRRK2 gene in sporadic Parkinson’s disease

Evidence supporting the theory states that LRRK2 non-coding variants influence disease risk via LRRK2 protein expression raises. For instance, it has been demonstrated that common variations at the LRRK2 locus induced more elevated levels of LRRK2 gene expression in human microglia-like cells (hiMGLs) emanated from monocytes [112]. Expression of LRRK2 increases transcriptional, which leads to the activation of inflammatory responses, and PD-linked mutations cause activated microglia to produce cytokines [113, 114]. LRRK2 inhibition, either via RNAi knockdown or small-molecule kinase inhibitors (SMKIs), reduces inflammatory responses in microglia, but otherwise, LRRK2 deficiency leads to impaired immune clearance in vivo [86, 114].

### LRRK2 genetic in idiopathic Parkinson’s disease

Concerning PD, each patient is diverse as clinically diagnosed, which is related to the different ages onset, the disease’s progression rate, and neuropathology. Among the diagnosed cases, only 10% are related to heredity gene mutation, and the remaining 90% are sporadic or idiopathic [115, 116]. The common variants in the LRRK2 locus pose a risk for idiopathic Parkinson’s disease (IPD) and claim that LRRK2 performs as a link between idiopathic and genetic PD [62]. LRRK2 kinase activity is notably boosted in microglia and SN dopamine neurons of patients with IPD, despite its inferior in abundance neurons [117]. Sustained elevation of LRRK2 kinase activity conceivable plays a part in the pathogenesis of IPD: Several researchers have established that LRRK2.

silencing or LRRK2 kinase inhibitors can prevent PD-related brain pathologies in rodent models [118–121]. Although it is becoming increasingly accepted that IPD is associated with increased LRRK2 kinase activity, the mechanism by which this protein is activated and its roles in the disease’s pathogenesis remain a mystery [122].

Patients with late-onset IPD and whose carriers of LRRK2 mutation develop PD are clinically identical. Notwithstanding clinical similarities, neuropathology is very heterogenous in the substrate of LRRK2-PD, precise among family members with the exact mutation [123]. Data demonstrated that PD patients with LRRK2 mutations indicate locus coeruleus and profound nigrostriatal degeneration. Regardless, there are varying degrees of Lewy pathology: while most G2019S carriers have Lewy bodies (LBs) pathology, a few do not. This variation among individuals with the same mutation implies that the pathology of α-Syn is not critical for the typical clinical features [64, 124].

## LRRK2 pathologies

### Autophagy and ubiquitin proteasome pathway

Defective autophagy leads to the expansion of misfolded proteins and sets the scene for neurodegeneration. Since neurons examine a post-mitotic state, they rely more on autophagy pathways to clear their cellular waste than other somatic lines [125]. Macroautophagy, microautophagy, and chaperon-mediated autophagy (CMA), are the three primary formats of autophagy specified so far. Macroautophagy is a complex process beginning with forming a cup-shaped membrane to engulf the aimed waste and then fuse with the lysosome [126]. Selective macroautophagy leans on cargo receptors that identify targeted elements and conscript them to autophagosomes, and between these, microtubule-associated protein 1-light chain 3 (known as MAP1LC3 or LC3) plays the leading role [127, 128]. CMA takes a more straightforward path and is just required to maintain the KFERQ-like motif.

The proteins are identified by cytosolic Hsc70, conducted to the lysosomal membrane, and finally attached to LAMP2A, a transmembrane receptor. Microautophagy is the least demanding form of autophagy to work. Substrates of the targeted particles are instantly eradicated by the lysosomal membrane, with no requirement for an intermediate autophagosome formation. The research showed a significant decline of lysosomal and CMA characteristics, such as LAMP1, LAMP2A, Hsc70, or Cathepsin D, associated with an expansion of autophagic identifications such as LC3II and p62 levels, the whole-brain but, the role of especially LRRK2 is to be discussed here [129]. Phosphorylating EndoA at Ser75 by LRRK2 controls autophagy and modulates the membrane curvature, thus regulating the recruitment of the autophagy machinery to the aborning autophagosome [37]. It makes clear sense that these vesicles are highly defined in macroautophagy. To do more, LRRK2 conducts more KFERQ peptide motifs in its amino acid sequence to be accessible for hsc70 targeting. Likewise, increased levels of WT or LRRK2 mutants impede the construction of the CMA translocation complex at the lysosomal membrane, consequently blocking CMA.

At last, LRRK2 affects autophagy by altering calcium levels. The role of calcium in autophagy induction has long been determined. TPC2 is expressed on lysosomes, and LRRK2 induces TPC2 and Ca^2+^ clearance from lysosomes, which initiates additional Ca^2+^ release from ER and generates autophagy via CaMKK/AMPK [130].

Earlier, we discussed the role of CMA, but many proteins play an essential role in another cellular protein correction/clearance strategy: the ubiquitin–proteasome pathway. First, misfolded proteins are assigned for UPS elimination. Preferably, the chaperone Hsp70 and the ubiquitin ligases E1, E2, and E3 mark misfolded proteins by expanding ubiquitin motifs on lysine residues. The subsequent stage proceeds by ubiquitinated misfolded proteins, which the 19S proteasomal subunit recognizes to eradicate ubiquitin chains with specific deubiquitinating enzymes [131, 132]. Eventually, the misfolded protein unfolds and is destroyed by the proteolytic 20S subunit in small peptides. Numerous structurally defective LRRK2 variants have the essence and stabilizing role of chaperons, which can be considered an alternative to PD therapies [133]. A particular heat shock protein, the C-terminus of Hsp70-interacting protein (CHIP), is specifically responsible for attaching to LRRK2 so E3 ubiquitin ligase can act on it and prevent LRRK2 aggregation. Distinct analyses have recognized the chaperone Hsp90 between the proteins connected with LRRK2. Hsp90 is paramount for retaining the LRRK2 protein’s strength and steady state of LRRK2 proteins. Disturbance of Hsp90 activity stimulates LRRK2 G2019S proteasomal degradation and leads to neural toxicity [134, 135].

### Cytoskeletal dynamics

Neurons and neural systems are all about connection, whether from the somata to the neural process or in the synaptic space. Also, dendrite-axon growth and remodeling depend on a trafficking system. Many intracellular/extracellular transportation depend on vesicles, but these vesicles need some tracks to move or complete their tasks. In addition to the discussed kinase/GTPase activity of LRRK2, the gene is also a domain for protein-to-protein interaction where an additional scaffolding of cellular components (cytoskeletal systems and vesicles) occurs [136]. Specifically, we can discuss the role of LRRK2 in two categories: LRRK2 and microtubules, LRRK2, and actin fibers.

MTs are polymers of α and β-tubulins, doing a significant portion of neuronal homeostasis, including neurite outgrowth, multidirectional transportation, and synaptic plasticity. Rudimental investigations indicated that the LRRK2 controls microtubules via its ROC domain [137]. It was reported that the G2019S mutation was aligned with increased phosphorylation; this phosphorylation enhances the MT stability [138]. Another study in LRRK2 knockout demonstrated increased MT acetylation. The same authors later prepared more proof for their work by finding more acetylated alpha tubulins in the kidney of LRRK2 knockout models employing an impartial high-throughput proteomic screen [139]. In line with this analysis, another study informed that R1441C and Y1699C LRRK2 mutants deface axonal transport in primary neurons in the Drosophila model, which shows raising the binding with MTs. However, the authors uncovered that mutant LRRK2 binds deacetylated preferably than acetylated MTs in-vitro [140]. Regarding actin, a REM protein called myosin attaches actin fibers to the plasma membrane. LRRK2 phosphorylates myosin and, therefore promotes actin polymerization [31].

### Inflammatory processes

There have always been speculations about the inflammatory triggers of PD. One of the most simple, most substantial facts helping this hypothesis is the old suggested pathway of dead dopaminergic cells which results in a debris-uptake of the immune system and other inflammatory responses to dead cells [141]. However, LRRK2, in particular, has been linked to the inflammatory nature of PD for various reasons. Apart from PD, LRRK2 has been a hot topic of discussion for autoimmune diseases such as IBD, type 1 diabetes, rheumatoid arthritis, celiac disease, psoriasis, and multiple sclerosis, which LRRK2 modulates multiple pathways in innate and adaptive immunity by hindering NF-κB and NF-AT signaling [142]. In addition, LRRK2 expression has been correlated with cytokine secretion levels indicating an immunoregulatory role for the gene. Activated LRRK2 suppresses c-Rel, and enables the formation of tau oligomers, which leads to cell extinction and an expansion in membrane receptors of inflammatory cytokines [143]. Finally, LRRK2 expression is higher in immune cells such as monocytes, microglia, and dendritic cells. In turn, pharmacological inhibition of LRRK2 in models prevents α-Syn-mediated neurodegeneration and reduces inflammation by a decrease in the activation of microglia [144, 145].

### Mitochondrial dysfunction

The role of mitochondrial dysfunction, which leads to higher oxidative stress, reduced mitochondria membrane potential, decreased ATP production, mitochondria DNA damage, mitochondrial elongation, mitochondrial fragmentation, and mitophagy in neurodegenerative disease, has long been discussed. However, a strong correlation between PD pathogenesis and mitochondria, specifically LRRK2, has recently been a spotlight for researchers to work on [146, 147]. Almost 10% of LRRK2 is attending in cells mitochondrial fraction, and the mitochondrial complex I activity is declined by 40% in the substantia nigra, the famous suspected area to account for PD since the beginning of investigating PD’s pathology [148]. Furthermore, in various postmortem human tissues from LRRK2-linked PD cases, other measures of G2019S-LRRK2 PD have been observed with different mitochondrial dysfunctions. We will look at how LRRK2 participates in these dysfunctions [146].

#### Mitophagy

Mitophagy refers to a mitochondrial mechanism that enables the degradation of sabotaged mitochondria and maintains cellular hemostasis in response to stress. However, discovering PD’s familial genes, PTEN-induced putative kinase 1 (PINK1), and Parkin (PRKN) in regulating mitochondrial degradation reconfirmed the strong correlation between this pathway of pathology and PD. Recently, it was evidenced that PINK1 and PRKN interact with LRRK2, and subsequently, we can define a role for LRRK2 in the mitochondrial dysfunction of PD patients [149]. Marking the outer membrane of mitochondria by PARKIN to isolate the impairment in mitochondria is one of the premature scenes of mitophagy and is facilitated through LRRK2 [148]. LRRK2 over-activation through the kinase enzymatic disrupts the interaction between Parkin and subunits of the outer mitochondrial membrane, such as Drp1 and MiD5. Furthermore, LRRK2 can phosphorylate RAB10, depolarizing mitochondria and making it a better substrate for the ubiquitin-binding adapter OPTN [150].

#### Mitochondria and calcium hemostasis

PD-susceptible SNc DA neurons need calcium ions to activate calcium-dependent voltage gates and make an electrical pace. The low affinity of mitochondrial calcium uniporter (MCU) plays a significant role in keeping the cellular hemostasis calcium [151]. Some suggest continuous high calcium levels in these neurons result in an excess ROS production for mitochondria to depose and create higher chances of cell death [152]. LRRK2, in particular, targets the principal presynaptic Ca influx pathway, the CaV2.1 channel, and is a defining factor in the calcium regulation of the cell [153]. WT-LRRK2 or G2019S-LRRK2 overexpression in-vitro cells have been declared to trigger the nicotinic acid adenine dinucleotide phosphate (NAADP) receptors to generate Ca^2+^ efflux from lysosomes again to alter cellular levels of calcium [154]. LRRK2-G2019S iPSC-derived sensory neurons indicate lessened Ca^2+^ reactions to KCl depolarization. It was shown that G2019S responds well to LRRK2 kinase inhibitors [155].

#### Mitochondrial dynamics

Mitochondria are dynamic organelles, frequently examining fission and fusion. Since GTPases and WD40 repeat dominantly control this turn-over, LRRK2 possessing both domains can act as a scaffold for the process [149]. LRRK2-G2019S knock-in mice present evolved mitochondria transformations compatible with halted fission [156]. Further, human dopaminergic neuroblastoma cell line SH-SY5Y expressing LRRK2 mutants (G2019S and R1441C) displays intense mitochondrial fragmentation with more concise and slighter mitochondria, which obeyed in electron micrographs [157].

### Lewy bodies and protein accumulation

**Fig. 3:**
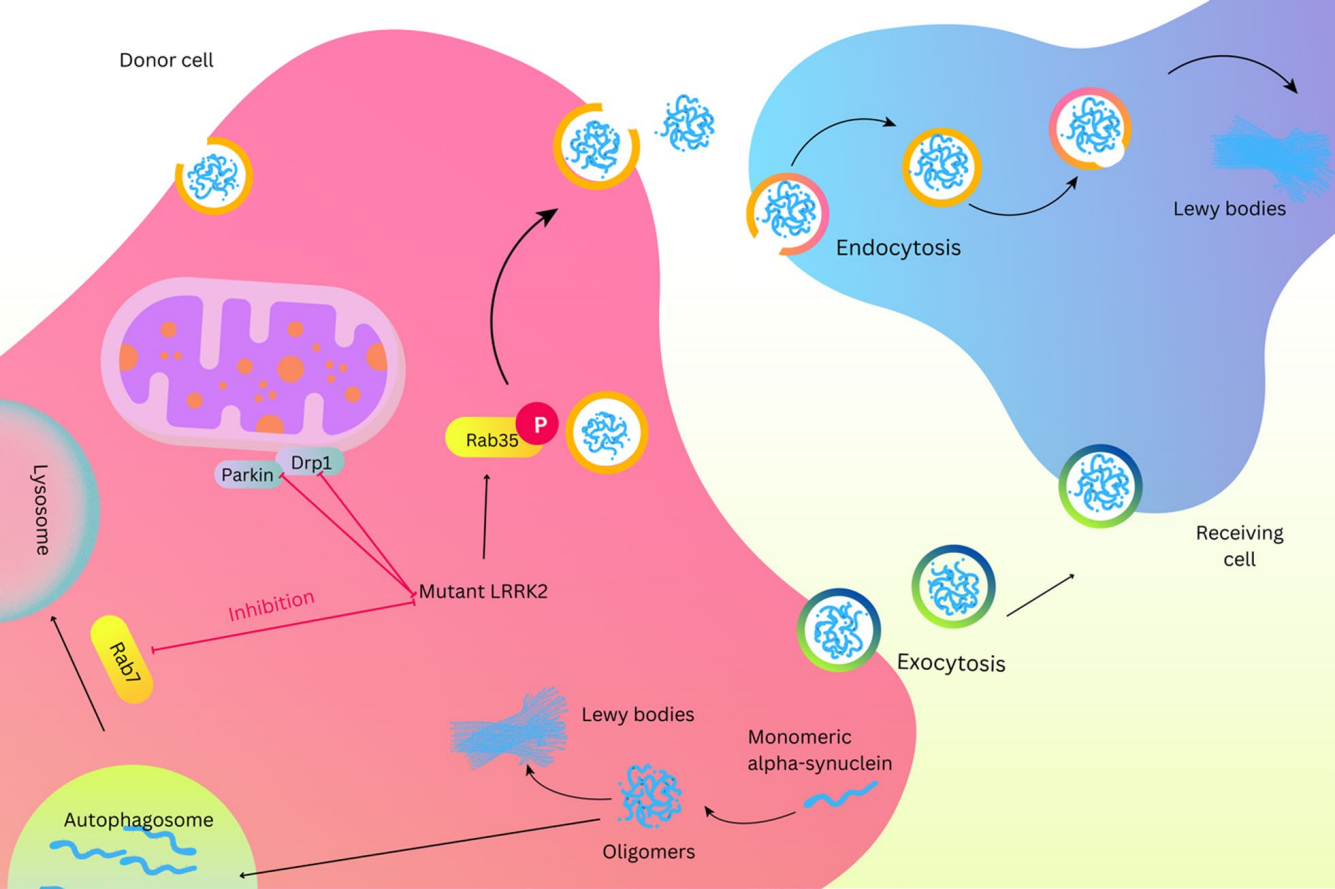
Mutant LRRK2 leads to the transmission of α-synuclein from one neuron to another. Mutation in LRRK2 stimulates kinase activity and phosphorylates Rab35, which involves α-synuclein released into the extracellular area by exosomes. These exosomes get into the cytosol of other neurons. Also, Rab7 inhibitions eventually prevent the endosome-lysosomal degradation of α-synuclein aggregates

Friedrich Heinrich Lewy first described the Lewy bodies (LBs) in 1912 [158]. LBs are an aberrant accumulation of insoluble proteins associated with misfolding and phosphorylated α-Syn (Fig. 3), malformed organelles, and a component of the lipid membrane associated with a group of disorders called Synucleinopathies. Synucleinopathies include PD, dementia with Lewy bodies (DLB), progressive nuclear palsy (PSP), multiple system atrophy (MSA), frontotemporal lobar degeneration (FTLD), and Alzheimer’s disease (AD) [159].

By ordinary physiological conditions, α-Syn monomers found in the cytosol of neurons or located near the presynaptic membrane, where they bind to the soluble N-ethylmaleimide-sensitive factor attachment protein receptor (SNARE) and regulate the SNARE membrane protein synaptobrevin-2 associated with vesicle 2 (VAMP2) [160, 161]. Underneath particular stress conditions, genetic mutation, or other undiscovered reasons, α-Syn aggregates into oligomers, subsequently forms fibrils and finally settles in the constitution of LBs. There is a hypothesis that neurons form these LBs for neuroprotection, as smaller clusters of α-Syn, notably oligomers, are more toxic [160, 162]. In these parts, we discussed that LRRK2 and α-Syn might be related during PD [163]. LRRK2 and α-Syn direct interaction include physical contact, which enables one to directly regulate the other’s function. Data displayed LRRK2 co-localization with phosphorylated α-Syn in human PD brain samples. According to this point that LRRK2 is a serine-threonine kinase, the desire of LRRK2 to interact with phosphorylate α-Syn is influenced by the G2019S-LRRK2 mutation, resulting in α-Syn accumulation and, ultimately, cell death [164–166].

Studies authenticated that A53T does not stimulate kinase activity; this was attained by deletion of the LRRK2 gene revved pathological features in A53T α-Syn/LRRK2 transgenic mice compared with A53T α-Syn/wild-type (WT) LRRK2 mice [163, 167].

In a similar study, Daher et al. [168] created a model of LRRK2 expression in an A53T α-Syn transgenic mice model. The result displayed that deletion of LRRK2 or overexpression of G2019S-LRRK2 has a minor influence on the lethal neurodegeneration phenotype that develops in A53T α-Syn transgenic mice. Also, the study showed that endogenous or human LRRK2 and A53T α-Syn do not interact concurrently to affect the number of nigrostriatal dopaminergic neurons. Tezuka et al. [169] declare an autopsy patient carrying the LRRK2-G2385R, a risk variant for PD. At the early stage, the patient displayed levodopa-responsive parkinsonism. The pathological investigation uncovered spread Lewy bodies with amyloid plaques, which induced neuro-inflammation. The study demonstrated that G2385R seems to raise LRRK2 activity in the human brain, generating dangerous circumstances in the brain area [169]. Of course, there is no convergence in all studies, so some have indicated that the phosphorylated α-Syn is diminished or untouched in mice expressing mutant LRRK2, implying that α-Syn maybe is not a substrate for the LRRK2 activity in vivo. This implies that no substantial evidence supports the direct phosphorylation of α-Syn by LRRK2. Therefore, LRRK2-linked PD patients do not show Lewy pathology. However, they may have an aggregation of other proteins, such as tau and TDP-43, proposing that PD due to LRRK2 dysfunction may come about unassisted by α-Syn accumulation [170–172].

## Affecting other genes

The subsequent significant inquiry is about the effects that LRRK2 has on cells and the organism. If we make the supposition that LRRK2 has some more elevated association with other hereditary types of PD, we could discover certain contenders for LRRK2’s cell function. The SNCA is the primary gene cloned for inherited PD, encoding small protein α-synuclein. The increased level of α-synuclein is a pleomorphic risk locus for PD and contains gene multiplications (duplications or triplications), point mutations, and risk factors for sporadic PD [173]. As declared before, quite an appealing idea is that PD may occur due to the defeat of degradative pathways for vesicular proteins, which influence neurons’ recycling of vesicles and the proteins associated with synapses [174]. It is shown that the activity of CMA decreases with age and that α-Syn protein stability increases with age and mutations [175, 176]. A conceivable justification for the age-dependent penetrance of LRRK2 mutations is that protein levels have a crucial role in toxic circumstances in the brain, assuming that the accumulation of mutations in SNCA increases protein levels and aggregations. Numerous PD-linked genes have been identified since the initial SNCA cloning, convergent on the vesicle trafficking pathways and autophagy-lysosome system. Pivoting on the evidence that discourses the role of LRRK2 in vesicle uptake and recycling, Table 1 summarizes several of the primary characteristics of these intracellular functions [174, 177, 178].

**Table 1:** PD-related genes with a role in organizing the endomembrane trafficking

Gene	Inheritance	Location	Function	References
Parkin	AR	6q25	• E3 ubiquitin ligase of damaged mitochondria for degradation via mitophagy	[190]
PINK1	AR	6q25	• Phosphorylation of mitochondria for mitophagy and parkin activation	[290]
Vps35	AD	16q13-q21	• Component of the retromer complex • LRRK2-interacting protein • The D620N mutation increases LRRK2 kinase substrate phosphorylation	[291, 292]
GBA	Risk factor	1q21	• Lysosomal protease • Autophagosome component	[26, 293]
SNCA	Risk factor/AD	4q21	• Pathogenic α-synuclein • Substrate of CMA • Inhibits CMA • Promotes macroautophagy	[15]

*AD* autosomal dominant, *AR* autosomal recessive

## LRRK2 role in the immune system

Despite LRRK2’s ubiquitous expression, exceptional mRNA and LRRK2 protein levels exist in lymph nodes, peripheral blood mononuclear cells (PBMCs), primary microglia, and the spleen [179]. CD14+ neutrophils, monocytes, CD19+ B cells, CD8+ T cells, and CD4+ T cells, healthy control PBMCs were increased all express LRRK2; although, expression is most elevated in CD16+, CD14+ neutrophils, and monocytes [180–182]. Stimulation with IFNγ or lipopolysaccharide (LPS) upregulates LRRK2 expression in myeloid cells such as first monocytes, human monocytic leukemia cell line (THP-1), or bone marrow-derived macrophages (BMM) [183–185]. In PBMCs of sporadic PD patients, the level of LRRK2 expression in CD16+ monocytes, T cells, and B cells are higher compared with healthy controls, and the connection between inflammatory activity and monocyte LRRK2 levels is considerably different in patients’ PD [186]. There is no expressive explanation of LRRK2 protein function in the immune system and how this can lead to disease pathogenesis [187]. Some suggest that LRRK2 may influence the maturation and activation of immune cells. These further eliminate the radical burst against pathogens in macrophages and modulate neuro-inflammation via cytokine signaling [145, 188, 189]. In Crohn’s disease [190], Like PD, IFN-γ-induced inflammatory gene expression and LRRK2 are present in immune cells [191]. Upregulated LRRK2 mRNA is in inflamed CD intestinal tissue compared with uninflamed tissue from a similar patient, and LRRK2 gene expression stood localized to dendritic cells, B cells, and macrophages in the lamina propria [183].

According to a study, human CD14+ monocytes from the blood cells of CD patients with the LRRK2 M2397T polymorphism indicate a raised pro-inflammatory response to IFNγ comparable to the control group [191–193]. It is increasingly appreciated that dopaminergic neurons of the substantia nigra (SN) are preferentially susceptible to degeneration in PD; hence, these neurons are more susceptible to the inflammatory response effects. Compared to the rest of the nervous system, adult mice have demonstrated a greater density of resting microglia in the SN (which accounts for approximately 12% of cells in this region) [194, 195].

Moreover, microglia stimulation can present in disease pathogenesis by releasing anti- or pro-inflammatory cytokines as microglia moderate the immune responses in the CNS, subsequently activating other mediators of repair, inflammation, and growth. Numerous researchers have appointed LRRK2 as the critical role of pro-inflammatory response downstream signaling elements. Recent research indicates that LRRK2 plays a role in phagocytosis, and increasing the LRRK2 protein level may inhibit the inflammatory response [186, 196, 197]. In vitro studies demonstrate that LRRK2 expression is enhanced in microglia clusters and bone marrow-derived macrophages after presenting pro-inflammatory agents like LPS. Furthermore, LRRK2 knockdown improves this inflammatory signaling, showing that inflammation and LRRK2 have a modulatory, complex relationship that still demands mechanical clarification [145, 185, 197, 198]. A more detailed link between neuroinflammation and the role of LRRK2 in PD has been discussed lately by Greggio and colleagues [199, 200]. The reality is that the LRRK2 gene expression in human PBMCs is high under homeostatic conditions, and increased LRRK2 protein levels after stimulation exhibit its induction plays a crucial part in regulating effector functions; hence, Caution should be exercised in targeting LRRK2 as an essential therapeutic intervention in PD as up to now imprecise whether the raised LRRK2 protein levels in immune cells are deleterious or protecting function in the immune system [201, 202].

## LRRK2 targeted therapy in Parkinson’s

### Drug discovery

Different mechanisms involved in PD pathology can be directly and indirectly related to LRRK2. Upstream inhibitors and up regulators of LRRK2 include (peroxiredoxin 2 (Prx2), SP1, and Fbxl18) and (HOTAIR and MALAT1) respectively [203, 204], and also interacting factor (TXNIP) affect LRRK2 regulation and lead to the following downstream factors ended with autophagy and lysosomal dysfunction, inflammation, vesicle trafficking, the deficiency of protein synthesis, mitochondrial dysfunction, oxidative stress, ER stress. Therefore, targeting LRRK2 for PD treatment can be separated into two approaches, direct and indirect suppression of LRRK2. Direct inhibition includes reducing LRRK2 activity (kinase and GTP-binding activities) and decreasing the total protein levels. Indirect inhibition includes inhibiting upstream and downstream factors, signaling pathways, and increasing LRRK2 antagonists [205]. There are many in-vivo and in-vitro studies according to drug discovery for PD based on LRRK2. To introduce new groups of LRRK2 inhibitor groups, the synthesis of macrocyclic molecules by linking the two terminal nitrogen atoms of HG-10-102-01 with an alkyl chain ranging from 2 to 4 units, was designed. This effort resulted in a cluster of molecules of both biochemical efficacy and BBB permeability, 9 out of 14 synthesized candidates demonstrated potent low Nano-molar inhibition and significant BBB penetration [206].

#### LRRK2 in-vitro model

An in-vitro study assessed the efficacy of LRRK2 inhibitors for IPD patients without coding sequence mutations in LRRK2. Induced pluripotent stem cells (iPSCs) were provoked by IPD patients. All iPSCs had two PD risk variants (SNCA and MAPT); the study group had LRRK2-RS1491923 risk allele C/C, but the control group had a non-risk T/T variant. Neurons with the C/C-risk allele showed nuanced autophagy and mitochondrial clearance distinctions compared to controls. It was also suggested that CZC-25146, as an LRRK2 inhibitor, can reduce α-Syn, a factor of pathology in all IPD lines [207]. Another in-vitro study compared iPSCs derived from PD patients and carried different mutations like LRRK2 with the healthy control. This study evaluated mitochondrial responses by assessing mitochondrial respiration, proton leakage, and ROS production. It was suggested that mitochondrial dysfunction is an existing factor leading to PD. Also, it was observed that coenzyme Q_10_, rapamycin, or the LRRK2 kinase inhibitor GW5074 ameliorated PD pathogenesis [208]. Mutant G2019S-LRRK2 mouse neurons were treated with LRRK2 kinase inhibitors, PF-06447475 and MLi-2 showed the inhibitory effects on G2019S-LRRK2 and the progression of pathological-Syn inclusions [209]. To re-examine MLi-2 inhibitory values, stroke-induced mice showing significantly increased LRRK2 activity in Rose-Bengal (RB) photo-thrombosis were examined. Results showed co-localize high activity of LRRK2 with mitophagy-related proteins and that MLi-2 could attenuate mitochondrial apoptosis, ultimately leading to neuroprotective potential in stroke progression [210].

Cui et al. (2021/China) performed an in-vitro study on the Homosapien bone marrow neuroblastoma (SH-SY5Y) cell line to assess the absorption rate of VB12, an inhibitor of LRRK2 activity, as VB12-loaded tetrahedral framework nucleic acid (TVC) by flow cytometry. Compared to free VB12, better autophagy recovery and higher absorption were observed [211]. Another study was done on the same cell line. It showed that treating the cell line with 6-hydroxydopamine (6-OHDA)-induced neurotoxicity by hesperidin (HES) can decrease catalase, superoxide dismutase, glutathione as efficiently as LDOPA and produce neuroprotective effects [212]. SH-SY5Y cells were investigated in another study. Based on the findings, expression of the PD-related mutant, LRRK2-R1441C, causes mitochondrial defects and lysosomal transport in the neurites of SH-SY5Y cells. More importantly, GTP-binding inhibitors can reduce LRRK2 GTP-binding activity and alleviate R1441C-induced mitochondrial and lysosomal transport disorders. These results indicate the primary evidence and mechanism for neurite damage that underlies LRRK2-induced neurodegeneration. Hence inhibition of LRRK2 GTP-binding could be a potential new strategy for PD intervention [213, 214]. Also, there is a relationship between glucocerebrosidase and LRRK2 activities in astrocytes. Lysosomal dysfunction and inflammatory responses caused by beta-glucocerebrosidase 1 (GBA1) mutations, a risk factor of PD, can be normalized by LRRK2 inhibitors [215, 216]. In other in-vitro study sed, GBA1-heterozygous-null iPSC-derived neurons instead of mutant GBA1 were used, and a decrease of glucocerebrosidase in DA neurons derived from LRRK2-PD patients with G2019S or R1441C mutations were observed [217]. A study by Kedariti et al. (2022/ Italy) showed the reduction of GCase protein level in G2019S iPSC-derived neurons. However, a positive relationship existed between GCase activity and LRRK2 kinase in patient-derived fibroblasts and peripheral blood mononuclear cells [218]. LRRK2 exon 41 skippings can decrease LRRK2 kinase phosphorylation and can also affect the autophagic process of LRRK2. So, LRRK2 exon 41 skippings can be considered one of the possible therapeutic strategies in PD [219]. LRRK2 can be inhibited indirectly through signaling pathways. Some in-vitro studies suppressing up regulators of LRRK2 (HOTAIR and MALAT1) can attenuate LRRK2-related PD [203, 204]. According to some pre-clinical studies, suppression of downstream mechanisms can be the other therapeutic target. One strategy is reducing MIRO**,** mitochondrial protein, levels identified as a molecular signature for detecting PD. The decrease in this protein can degrade damaged mitochondria [220]. The second strategy is to inhibit pancreatic endoplasmic reticulum kinase (PERK), which can prevent Tau-mediated neurodegeneration (like PD) in a mouse model [221]. SERCA is another potential target. LRRK2 deactivates SERCA leading to mitochondrial dysfunction and ER stress [222]. Antisense oligonucleotides (ASOs) are a conceivable technique to decrease the total protein levels of LRRK2. Furthermore, some pre-clinical evidence showed that ASOs reduced α-Syn, whose relation with LRRK2 was mentioned before [223]. An in-vitro study on COS-7, HeLa, and SH-SY5Y cells showed that over-expression of Prx2, an LRRK2 inhibitor, decreased LRRK2 activity and LRRK2 mutant-induced apoptotic processes [224]. Fbxl18, another upstream inhibitor of LRRK2, can be a potential target to prevent cell death caused by LLRK2 and PD-related mutant LRRK2 [225]. To assess RN277 and RN341’s ability to inhibit LRRK2 kinase activity towards its physiological substrates in vitro, a series of incubated dilutions of the compounds with recombinant LRRK2RCKW and Rab8a, and quantified Rab8a phosphorylation (pT72) were tested by mass spectrometry. The obtained results indicated increasing quantities of each compound followed by a decrease of Rab8a phosphorylation, indicative of inhibited LRRK2 kinase activity [226] to test OPM-38 as an LRRK2 inhibitor, SH-SY5Y neuroblastoma cells were transfected with hG2019S or hWt LRRK2, manifesting colon carcinoma. OPM-383 displays good permeability, metabolic stability, and capability to cross the blood–brain barrier with favorable drug-like properties [227]. In a more recent study, patient-derived GBM cells were orthotopically transplanted into mice models. This work introducing DNK72 as a novel LRRK2 inhibitor demonstrated that LRRK2 inhibition with DNK72 effectively reduced tumor growth and increased survival time [228].

#### LRRK2 in-vivo model

One in-vivo study injected wild-type and transgenic G2019S-LRRK2 rats with human α-synuclein intracranially. Afterward were treated with PF-06447475 in the study group or the control group with a compound for 4 weeks. It is reported that rats expressing G2019S-LRRK2 exhibited dopaminergic neurodegeneration because of α-Syn overexpression. Furthermore, LRRK2 kinase inhibition was observed to be useful for neuroinflammation and neurodegeneration [121]. In another in-vivo study, 50 mice were divided into 5 groups, including tetrahedral framework nucleic acid (TFNAs) group, TVC group, VB12 group, 1-methyl-4-phenyl-1,2,3,6-tetrahydropyridine (MPTP) group, and control group. Controls received normal saline, and mice in other groups were treated with MPTP additionally. TEM images of different organs of mice showed that TVC had better absorption in the brain. Moreover, the TVC group showed better clearance of abnormal protein accumulations than free VB12, which attenuates the motor syndrome of PD [211]. Ogier et al. (2020/Australia) suggested that ASK1 (a downstream factor of LRRK2) knockdown improves behavioral therapy in neuro-damaged mice combined with oxidopamine [229]. Also, an in-vivo study was performed on a Drosophila model to understand the relationship between the MAPK pathway and LRRK2. The result showed that LRRK2 activated JNK resulting in neural death and motor damage, So JNK inhibitors can be considered a therapeutic PD strategy [230]. In a recent study, mice were injected with tau fibrils and treated with a control diet or diet containing LRRK2 kinase inhibitor MLi-2 targeting the IC50 or IC90 of LRRK2 for 3–6 months, and systemic alterations in the progression of tau pathology were seen consistent to prior studies done by the team [231]. LRRK2 kinase inhibitors which resulted in the introduction of a new animal model for testing the disease. Since LRRK2 was necessary for the development and regeneration of Nematostella, A newly introduced LRRK2 inhibitor, PF-06447475 (PF-475), tested on female Drosophila melanogaster flies suffering from paraquat-induced locomotor impairment, showed that this kinase inhibitor can retain dopaminergic activity, LPO activity and increase the overall lifespan of these animals [232]. PF-475 was also tested on Murine models with relapsing–remitting Multiple Sclerosis to see whether it has the ability to reverse the progressive demyelination through enhancing primary OPC proliferation and differentiation. The results showed that PF-475 exerted a statistically significant reduction of the clinical burden of the animals, and histological evidence revealed how the treated animals presented a reduced lesion area in the spinal cord [233].

#### Safety for LRRK2 kinase inhibitors in preclinical trials

LRRK2 is not only expressed in the brain. It can also be expressed in the kidneys, the lungs, and the immune cells [234]. So, the safety of LRRK2-targeted drugs should be investigated in preclinical and clinical trials.

##### Side effects of pharmacological LRRK2 inhibition on kidneys and lungs

LRRK2 knock-out rats and mice showed adverse effects in the kidneys and lungs. An in-vivo study assessed whether pharmacological inhibition of LRRK2 has the same results by oral administration of PFE-360 for 12 weeks. It showed kidney darkening and an advanced assemblage of hyaline droplets in the proximal renal tubules (but not the lung); It was while Renal tubular injury or the reduction of kidney function was not observed [235]. Another in-vivo study was conducted by MitoPark mice treated with MLi-2 at 30 mg/kg/day for 15 weeks. Following the treatment, there was a morphological change in the lungs as pneumocyte type II enlargement, and there was no kidney problem like darkening or renal tubule pigmentation [236]. One study on nonhuman primates showed that LRRK2 inhibitor treatment did not cause kidney problems [237]. An in-vivo study by Baptista et al. (2018/USA) administered GNE-7915 (30 mg/kg positive control), PFE-360 (3 and 6 mg/kg), MLi-2 (15 and 50 mg/kg) for 2 weeks. This study showed that a lower dose of PFE-360 and MLi-2 did not induce lung pathology. No higher or lower dose did not show pulmonary function impairment [238].

##### Side effects of pharmacological LRRK2 inhibition on immune cells

Immune cells have the most expression of LRRK2, especially in monocytes and neutrophils [181], and it is linked to mitochondrial function, autophagy/ lysosomal function, and regulating inflammation [239]. LRRK2 has different roles in the immune cells, such as maturation and function of monocytes, affecting immune signaling pathways, regulating inflammation, and also the clearance of pathogens [182, 240, 241]. Activation of NLRC4 inflammasome when the body is infected with intracellular bacteria is along with the remarkable response of the host. NLRC4 requires phosphorylation for activation, which correlates with LRRK2 kinase activity. By inhibition of LRRK2 via GSK2578215A (second-generation LRRK2 inhibitor), the clearance of *Salmonella typhimurium* is decreased [242]. Reversely, another study showed that murine macrophages treated with the same LRRK2 inhibitor could increase the clearance of *M. tuberculosis,* like LRRK2-knock-out mice [197]. Neuroinflammation can be increased by the elevated immune response due to LRRK2 mutation [243]. To find more side effects that may be caused by LRRK2 inhibitors better understanding of LRRK2 biology and further studies are required.

### Pharmacological approaches to targeting LRRK2 in PD

Substantial evidence suggests that LRRK2 interference with kinase activity correlates enzymatic processes with cytotoxicity in neurons cultured for over 15 years [244]. There has also been significant interest in academia and industry in developing a potent, brain-selective inhibitor of LRRK2 or other approaches such as gene silencing and targeted protein degradation (TPD) (Table 2). With recent studies, there is a need to expand the two main issues of molecules’ effectiveness in preclinical models and minimize the adverse effects of LRRK2 inhibitors in peripheral tissues, especially the lungs, and kidneys [245].

**Table 2:** Pharmacological approaches to target LRRK2 in PD

Approach	Type of molecules/method of modification	Mechanism of action	Origin	Model	Clinical stage	Outcome	References
Inhibition	Macrocyclic	LRRK2 kinase inhibitors	Synthesis	ADP-Glo kinase assay kit	N/A		[294]
Inhibition	HG-10-102-01 (a crystalline solid)	Inhibits Ser910, Ser935 phosphorylation	Synthetic organic	Endogenously expressed LRRK2 in human lymphoblastoid cells derived from control and Parkinson’s patient homozygous for the LRRK2 [G2019S] mutation	N/A		[295]
Inhibition	GSK2578215A	Induces autophagy	Synthesis	SH-SY5Y cultured cells	N/A		[237]
Inhibition	GNE-0877	Inhibits Ser1929 phosphorylation	Synthesis	Cynomolgus monkeys	N/A		[296]
Inhibition	FX2140	Increases LRRK2 ubiquitation through GTP-binding	Synthesis	IPCS-derived from patients with sporadic PD	N/A		[213]
Inhibition	BIIB122 (small molecule)	Inhibits Ser935 and T73 Rab10 phosphorylation	Synthetic	184 healthy individuals	Phase 1		[297]
Inhibition	**DNL201 **(small molecule)	Inhibits Rab10 phosphorylation	Synthetic	29 people with Parkinson’s disease	Phase1b		[298]
Degradation	Bifunctional compounds (degrader or PROTAC)	Degraders of wild-type and mutant forms of LRRK2, which consist of targeting ligand (an amino pyrimidine or indazole) that binds LRRK2, the degron, which represents a ligand that binds an E3 ubiquitin ligase, and the linker which has a moiety that connects the degron and the targeting ligand covalently	Synthesis	Cell lines used were mouse embryonic fibroblast (MEF) WT, LRRK2 homozygous knock-ins in MEFs [R1441C; VPS25N(D620N); G2019S]	N/A		[253]
Degradation	Bifunctional compounds (degrader or PROTAC)	Degrading LRRK2 using PROTAC strategy PF-06447475/CRBN-based PROTACs and the three GNE-7915/CRBN-based PROTACs	Synthesis	GFP-tagged LRRK2 transfected HEK293 cells were treated with DMSO, original kinase inhibitor and PROTAC	N/A	• MG132 $\uparrow$ ubiquitin signal • Indazole analogs $\downarrow$ levels of LRRK2 • Amino pyrimidine analogs $\uparrow$ levels of LRRK2	[247]
Gene modification	Zinc finger nucleases (ZFNs)	ZFNs designed to introduce a double-strand break adjacent to the G2019S mutation of the LRRK2	Synthesis	hiPSC-derived neurons	N/A	$\uparrow$ Extra cellular-signal-regulated kinase 1/2 (ERK)	[299]
Gene editing	CRISPR/Cas9	The combination of a 141-nt/ssODN and an editing cassette that targeted LRRK2 exon 41 was used to promote HDR of the Cas9-mediated double-stranded DNA break	Synthesis	Cell lines used were induced pluripotent (Cj-iPSCs) stem cells and marmoset embryonic (Cj-ESC)	N/A	Similar to humans, common marmoset’s LRRK2-G2019S mutation $\uparrow$ kinase activity	[300]
Gene correction	TALENS	TALEN-plasmids and a donor DNA plasmid designed targeting LRRK2 exon 41	Synthesis	Gene-corrected iPSC (LA5-GC TALEN: alternative name of stem cell line)	N/A	• DA neurons positive for S129P-αS can form neurites > 2000 μm • LRRK2-G2019S mutation prevents DA neurons from developing neurites longer than 2000 m	[301]
Gene correction	• Bacterial artificial chromosome (BAC) DNA episomal plasmids • pCXLE-hSK (KLF4, SOX2) • pCXLE-hUL (LIN28, L-MYC) • pCXLE-hOCT3/4-shp53 (shp53, OCT3/4)	BAC-based HR system for gene therapy of LRRK2-G2019S mutant iPSC produced via reprogramming episomal vectors	Synthesis	iPSC/Alternative name of stem cell line: SK-C-LRRK2-iPSC	N/A	The iPSCs had a normal karyotype	[302]

*PROTACs* proteolysis targeting chimeras, *NT* nucleotide, *ssODN* single-stranded oligodeoxy nucleotide, *HDR* homology-directed repair, *BAC* bacterial artificial chromosome, *iPSCs* induced pluripotent stem cells, *HR* homologous recombination

### TPD (targeted protein degradation) for LRRK2

Targeted Protein Degradation (TPD) represents a groundbreaking paradigm in drug discovery, exploiting the cell’s intrinsic degradation machinery, such as the ubiquitin–proteasome system (UPS) or autophagy, to selectively degrade specific proteins of interest (POIs) [246, 247]. This approach is particularly advantageous for targeting non-enzymatic and structural proteins that are otherwise inaccessible to traditional small-molecule inhibitors. These proteins, often implicated in the pathology of neurodegenerative diseases, include misfolded or aggregating species such as tau, α-Synuclein, and Huntingtin, whose pathological accumulation is central to diseases like Alzheimer’s, Parkinson’s, and Huntington’s, respectively [248]. Among the TPD strategies, Proteolysis Targeting Chimeras (PROTACs) is the most extensively studied and applied. PROTACs are heterobifunctional molecules that simultaneously bind the target protein and an E3 ubiquitin ligase, bringing the two into proximity. This proximity facilitates the ubiquitination of the target protein, marking it for degradation by the proteasome. First conceptualized in 2001, PROTAC technology has since evolved into a powerful tool for degrading proteins considered "undruggable" by conventional small molecules [249, 250]. While PROTACs utilizing UPS-mediated degradation have been transformative, their utility has limitations. UPS-mediated catabolism is biased toward proteins of lower molecular weight and those that can be efficiently ubiquitinated. As a result, many larger proteins or those with conformational constraints may escape degradation. Additionally, there is the inherent complexity of forming a stable ternary complex between the target protein, the PROTAC, and the E3 ligase—a process that depends on the precise structural compatibility of all three components [249, 250].

To overcome these challenges, alternative pathways like macroautophagy, which enables the degradation of larger cellular components, have been explored. In macroautophagy, targeted cellular components are sequestered into autophagosomes, which subsequently fuse with lysosomes, where their contents are degraded. This pathway could offer a complementary approach to PROTACs degrading large aggregates of misfolded proteins, such as those seen in PD [251]. LRRK2, a significant target in PD, is a prime example of a challenging protein for traditional kinase inhibitors. LRRK2 mutations, particularly the G2019S variant, are known to increase kinase activity, contributing to the pathogenesis of PD. Traditional small-molecule inhibitors of LRRK2, while effective in reducing kinase activity, have limitations due to their ATP-competitive nature, which can lead to off-target effects and inadequate degradation of the protein [251, 252]. Therefore, Kargbo et al. searched for patents connecting to the targeted degradation of LRRK2. PROTACs targeting LRRK2 typically consist of an LRRK2-binding ligand (such as an aminopyrimidine or indazole derivative), a linker, and an E3 ubiquitin ligase ligand. For example, compounds 1 and 3, while able to inhibit LRRK2 phosphorylation at Ser935 and Rab10, were initially found to be ineffective in promoting LRRK2 degradation. This observation underscored the critical importance of ternary complex formation, specifically the interaction between LRRK2, the PROTAC, and the E3 ligase, which may not have been adequately established [253]. Moreover, further studies suggested that modifications in the PROTAC’s linker region, including alterations in length, flexibility, and the attachment point to the ligands, could significantly enhance its ability to recruit E3 ligases and promote ubiquitination. A key limitation noted was that LRRK2 may not always be accessible to the E3 ligase component of the PROTAC, due to conformational or spatial restrictions that hinder the formation of a stable ternary complex. This insight highlights the ongoing need to optimize linker design and structural features of PROTAC molecules to ensure the efficient degradation of targets like LRRK2 [247]. Significant progress has been made in overcoming these challenges. Liu et al. [254] developed *XL01126*, a PROTAC that efficiently degrades LRRK2 by forming a highly cooperative ternary complex with the von Hippel-Lindau (VHL) E3 ligase. This degrader achieved robust LRRK2 degradation with a DC50 in the low nanomolar range, exhibiting a rapid and potent degradation profile that outperforms traditional kinase inhibitors. Hatcher et al. developed a PROTAC based on the MLi-2 scaffold, leveraging Cereblon (CRBN) for LRRK2 degradation. This compound not only inhibits LRRK2 kinase activity but also eliminates its scaffolding functions, addressing both enzymatic and non-enzymatic roles of LRRK2 in neurodegeneration [255]. These advancements demonstrate the evolving sophistication of PROTAC technology and its potential to deliver highly selective, potent, and multi-functional therapeutic interventions. As the design of PROTACs becomes more refined, with optimizations in linker flexibility, length, and attachment points, the ability to precisely degrade problematic proteins like LRRK2 will likely expand, offering novel treatments for PD and beyond. This continued progress underscores the transformative potential of TPD in modern medicine, providing a critical platform for the development of next-generation therapeutics aimed at previously unreachable targets.

### LRRK2-based biomarker assay

A biomarker for PD, as well as potentially a therapeutic target, is being studied for LRRK2. Elevations in LRRK2 or apparent changes in its phosphorylation state, i.e., pS1292-LRRK2, have been associated with the severity and progression of the disease. Thus, LRRK2 would appear to represent a good candidate for use in early diagnosis, prognosis, and monitoring of treatment response [256]. Researchers are developing highly sophisticated techniques—like immunoassays, Western blot, ELISA, and mass spectrometry—to measure LRRK2 along with its phosphorylated forms in biological fluids such as CSF, blood, and urine. An entirely new perspective on the issue is the analysis of urine exosomal LRRK2 phosphorylation. This noninvasive approach would enable the determination of mutation carriers, such as G2019S, and improve our prediction of the onset of disease and possible benefits of therapy. Blood biomarkers, such as LRRK2 kinase activity, are similarly under investigation for utility in monitoring disease progression and guiding personalized treatment strategies. All in all, there is a dual incentive in LRRK2 research concerning PD: as a target for innovative therapies and as a tool for purposing diagnosis and follow-up [257]. Though there remain challenges, the progress made thus far demonstrates the capacity to change the understanding, diagnosis, and treatment of PD. Much will depend on future research and clinical validation to deliver on this promise and better the lives of patients. The integration of LRRK2 as both a therapeutic target and a biomarker offers the dual potential to both treat and track the progression of PD, facilitating early intervention and the development of more personalized therapies [258].

LRRK2 pathway detection and quantification might be a practical tool for evaluating activation during the different stages, including early disclosure [259, 260]. LRRK2 detection can be used in long-term research to investigate disease advancement, analysis in clinical trials as an index of target conflict, and as a marker to anticipate feedback management [50, 261]. PD is a central nervous system disorder (CNS), but LRRK2 is not a soluble protein in cerebrospinal fluid (CSF) [262]. This point causes a challenge when a researcher extends an investigation [263]. Several studies have verified LRRK2 retrieval in CSF [264, 265]. The concept is based on exosome enrichment by ultracentrifugation and Western blot to distinguish total LRRK2 and pS1292-LRRK2 signals. Results have proved that CSF pLRRK2 was determined not to associate with disease severity and vice versa with urinary levels of pLRRK2 [266]. By the toxic kinase function assumption in PD and designing a test based on measuring the phosphorylation of LRRK2-S1292 in urine, we can show high levels of pS1292 in people with G2019S mutation [34]. The development of such assays needs perceptive and explicit anti-LRRK2 antibodies and Western blot analysis to verify the existence of phosphorylated LRRK2 in urinary exosomes [267–269].

Based on a particular tissue or cell evaluation, LRRK2 Levels could evolve, as proposed, expression levels are more affluent in the periphery, such as in blood cells [270, 271]. Blood-based biomarkers are admitted in various conditions, and identifying reliable biomarkers would be of exceptional value in the diagnosis as it is simple to manage [272]. Ongoing research has exposed some LRRK2 markers in the blood. These researches are referred significantly into the LRRK2 levels and function in blood for patient ordination or authorizing the assumption that PD patients without LRRK2 mutations have raised LRRK2 activity (especially kinase activity that leads to PD pathogenesis). The indeed broadly utilized for estimating LRRK2 kinase demonstrated the phosphorylation levels of LRRK2 on residue Ser935 via immunoassays [273, 274]. However, the levels of LRRK2 Ser935 could not be different between normal and PD patients. Therefore, attending the LRRK2 kinase inhibitor approach lessens its value as a biomarker [274]. LRRK2 phosphorylates several Rab GTPases, so antibodies that are phospho-specific developed to measure the LRRK2-dependent Rabs inhibit in PBMCs, especially pT73-Rab10, pS935-LRRK2, and pT73- Rab10 which have all been deliberated in PBMCs [275]. One must handle the frequently relevant cell types, such as monocytes, to identify LRRK2 characteristics [276]. Purified monocytes are the most applicable cell culture to explore when advancing blood-based markers of heightened LRRK2 pathway activity in the PD [180, 181]. Affirming the place where samples are collected and the type of research, several procedures are run for this aim: such as Western Immunoblots, ELISA [277], Mass Spectrometry [278], and Positron emission tomography (PET) [279]. Western blots are accepted to recognize and measure entire levels of LRRK2 from particular origins. This method for LRRK2 works based on attack pS935-LRRK2, pS1292-LRRK2, and pT73-Rab10 by antibodies [47, 280] or assessing the activation of LRRK2 kinase and its function. The further procedure is ELISA, which is used to accurately reveal the level of LRRK2 due to the disadvantages of Western blotting [47]. ELIAS is applied for LRRK2 kinase activity and pharmacodynamics of LRRK2 kinase inhibitors [281]. Liquid chromatography-mass spectrometry (LC–MS) is another detection technique [282] that the researcher adopts to evaluate overall LRRK2 and pS1292-LRRK2. The considerable disadvantage is that LC–MS runs based on recognizing a tiny part of peptide, which could not be definitive to LRRK2. Discussed approaches were based on the analysis of LRRK2 level, but PET imaging with a radiolabeled molecule can be manipulated to estimate that molecule’s bio-distribution [279, 283]. So approving the appraisal of brain infiltration is challenging in the clinical context. PET imaging can likewise be exploited to compute a verified CNS target conflict [284]. The testimony of an LRRK2 PET ligand could facilitate the clinical outcome of LRRK2 kinase inhibitors [285]. Rideout et al. recently reviewed the LRRK2 assay, biomarkers, and new methods [50].

## Targeting LRRK2 in clinical trials and challenges ahead

Various compounds are identified to inhibit LRRK2 and initiate a new drug discovery in PD. Up to now, 4 generations of LRRK2 inhibitors are considering clinical trials. The first generation (generation "0") are H-1152, GW5074, staurosporine, and sunitinib. They are non-selective and have poor potency. The second generation (generation "1") are LRRK2-IN-1, CZC-54252, CZC-25146, and TTT-3002, with improved selectivity but little brain penetration. The third generation (generation "2") are HG-10-102-1, JH-II-127, GSK2578215A, GNE-7915, GNE-0877, GNE-9605, PF-06447475 have higher selectivity and potency, and oral availability. However, their penetration is low to high, but they have a poor half-life. Finally, MLi-2 and PF-06685360 are the last generation (generation "4") compounds with higher potency and better penetration into the brain [35].

In the process of drug discovery, there are lots of challenges that researchers are faced. For instance, the biomarkers and measurement assays to LRRK2 activity must be increased. Measurement of phosphorylated LRRK2 on serine residues Ser910 and Ser935 needs to assess LRRK2 activity. While using an LRRK2 kinase inhibitor prevents the phosphorylation of these sites because it depends on LRRK2 kinase [39]. Another example is the relation between Rab10 phosphorylation and LRRK2 kinase activity; it was declared that the interplay between Rab phosphorylation and LRRK2 kinase activity is essential for regulating cellular signaling pathways and controlling various cellular functions. Dysregulation of these processes has been implicated in several diseases, including PD [36, 181, 234]. The other challenge is the selection of patients and timing. Patients who enroll in clinical trials should have LRRK2 mutations, which are rare in most countries and not epidemic. It is more pleasant to select the presymptomatic population of people with LRRK2 mutations to test the efficacy of drugs in delaying or preventing symptoms that can be counted as a challenge [286]. The other significant challenge is safety, which should be assured in clinical trials. The expression of LRRK2 is not limited to nervous tissue, and it can be expressed in the respiratory, immune system, and kidneys. So, it is expected to have different peripheral effects [287]. As was declared in the previous section, evidence showed some adverse effects in preclinical tests. Based on a study, it should be considered that LRRK2 mutations protect the body against pathogenic infections, so the absence of LRRK2 can increase the risk of infection [288]. Also, other adverse effects that may happen after chronic consumption of drugs or consumption by people with special conditions should be identified by post-marketing surveillance [289].

## Conclusion

The role of LRRK2 has proved as an essential protein in the pathology of PD. LRRK2 kinase disturbs different neural/inter-neural activities and leads to different pathogenesis pathways. According to studies, the diagnostic value of LRRK2 alone increases because LRRK2 isolation has been found not only in familial PD but also in sporadic forms of PD. There is already a significant number of biomarkers for studying PD. LC–MS techniques and mass-spectrometry continue to prove practical tools in building the future of sensitive, easy-to-use biomarker discovery, identification of Rab proteins as the substrate of LRRK2 activity, and Clinical diagnosis. We seek a more promising and instantaneous diagnosis of PD as more LRRK2-specific biomarkers become available in the next decade. Since the first effort to understand LRRK2 pathologies, mitochondrial dysfunction has the most supporting data for the defined mechanism involving LRRK2, but the distribution of mutant LRRK2 has been reported as heterogeneous in the faulty mitochondrial cells of the brain. So, there must be further investigation into non-neural cells’ role in mitochondrial dysfunction. The α-Syn-mediated neurotoxicity, as one mainstay of research fields in neurodegenerative diseases, has been strongly correlated with LRRK2, but it should be worked on its exact mechanism. Despite solid evidence of LRRK regulating inflammation in both central and peripheral immune systems, the exact mechanisms are yet to be defined. Recent exciting confirmations indicate an emphatic link between LRRK2 and cytoskeletal dynamics. Altered vesicular dynamics due to mutant LRRK2 can highly affect cell growth, cell repair, autophagy, and synaptic trafficking. Over 100 compounds with LRRK2 inhibitory features were introduced, and a good number showed desirable outcomes. According to the evidence, current studies should aim to narrow the gap between the efficacy of these drugs and adverse side effects in peripheral tissues, especially in the lungs and kidneys.

## Data Availability

Not applicable.

## Abbreviations

PD: Parkinson’s disease
LRRK2: Leucine-rich repeat kinase
MAPK: Mitogen-activated protein kinase
WDR: WD40 domains
DA: Dopamine
SNPc: Substantia nigra pars compacta
aSyn: α-Synuclein
MAPK: MAP kinase
SMKIs: Small-molecule kinase inhibitors
SNPs: Nucleotide polymorphisms
IPD: Idiopathic Parkinson’s disease
LBs: Lewy bodies
CMA: Chaperon-mediated autophagy
CHIP: C-terminus of Hsp70-interacting protein
PINK: PTEN-induced putative kinase 1
DLB: Dementia with Lewy bodies
PSP: Progressive nuclear palsy
MSA: Multiple system atrophy
FTLD: Frontotemporal lobar degeneration
AD: Alzheimer’s disease
LPS: Lipopolysaccharide
THP-1: Human monocytic leukemia cell line
BMM: Bone marrow-derived macrophages
HES: Hesperidin
GBA 1: Beta-glucocerebrosidase 1
PERK: Pancreatic endoplasmic reticulum kinase
TPD: Targeted protein degradation
UPS: Ubiquitin–proteasome system
PROTAC: Proteolysis targeting chimera
CNS: Central nervous system
PET: Positron emission tomography
LC-MS: Liquid chromatography-mass spectrometry
